# Proton pump inhibitors use and the risk of osteoporosis and fractures: A two-sample Mendelian randomization study

**DOI:** 10.1097/MD.0000000000049964

**Published:** 2026-07-24

**Authors:** Ping Li, Ruiji Wu, Hangchu Shi

**Affiliations:** a Department of the Operating Room, Zhejiang Provincial People’s Hospital (Affiliated People’s Hospital, Hangzhou Medical College), Hangzhou, Zhejiang, China; b Department of Orthopedics, Taizhou Hospital Affiliated to Wenzhou Medical University, Linhai, China; c Department of Orthopedics, The Third People’s Hospital of Yuhang District, Hangzhou, Zhejiang, China.

**Keywords:** bone mineral density, fracture, Mendelian randomization, osteoporosis, proton pump inhibitors

## Abstract

The causal relationship between proton pump inhibitor (PPI) use and bone health outcomes remains uncertain. This study employs a Mendelian randomization (MR) approach to investigate the potential causal association between PPI use and the risk of osteoporosis and fractures. We selected 4 representative PPIs, including omeprazole, esomeprazole, lansoprazole, and rabeprazole, for our study. Bone health outcomes were evaluated through femoral neck bone mineral density (BMD), lumbar spine BMD, and the prevalence of osteoporosis and fracture across various anatomical sites, including the upper arm and shoulder, wrist and hand, lumbar spine and pelvis, femur, and lower leg and ankle. To evaluate PPI exposure and bone health outcomes, we utilized summary statistics derived from genome-wide association studies conducted in European ancestry populations. Primary causal estimates were derived using the inverse-variance weighting (IVW) approach, supplemented by MR-Egger, weighted median, and Mendelian Randomization Pleiotropy Residual Sum and Outlier methods. To strengthen the robustness of our findings, we conducted sensitivity analyses encompassing assessments of heterogeneity, horizontal pleiotropy, and leave-one-out analyses. Lansoprazole demonstrated a significant positive causal effect on femoral neck BMD (IVW: β = 0.137, 95% confidence interval: 0.063–0.210, *P* = 2.73E−04), whereas no statistically significant effects were observed for lansoprazole on lumbar spine BMD, osteoporosis, or fracture risk. Esomeprazole showed a marginal causal association with an elevated risk of femur fracture (IVW: odds ratio = 1.049, 95% confidence interval: 1.004–1.096, *P* = .031); however, this association lost statistical significance following Bonferroni correction. Its effects on BMD, osteoporosis, and fractures at other anatomical sites remained nonsignificant. No causal associations with BMD, osteoporosis, or fracture risk were identified for either omeprazole or rabeprazole. Sensitivity analyses further reinforced the robustness and reliability of these findings. This MR analysis found no compelling evidence to support a causal association between PPI use and the risk of osteoporosis or fractures.

## 1. Introduction

Osteoporosis is a prevalent skeletal disorder characterized by reduced bone mass and increased bone fragility, significantly increasing susceptibility to fractures.^[1]^ Hip, vertebral, and wrist fractures are strongly associated with osteoporosis, contributing to substantial morbidity and mortality, especially among the elderly.^[2]^ In the European Union alone, an estimated 5.5 million men and 22 million women are affected by osteoporosis, resulting in nearly 3.5 million fractures annually.^[3]^ The prevalence, prognosis, and economic impact of fragility fractures in Europe and North America now rival, if not exceed, those of cardiovascular diseases.^[4]^ As the global population ages, the number of individuals affected by osteoporosis is projected to rise, imposing a growing economic strain on healthcare systems worldwide.^[5]^

Proton pump inhibitors (PPIs) are widely prescribed for the management of acid-related disorders, including gastroesophageal reflux disease and peptic ulcers, ranking among the most frequently dispensed medications globally.^[6,7]^ Nearly a quarter of the adult population worldwide uses PPIs.^[8]^ In 2022, omeprazole ranked as the ninth most prescribed medication in the United States, with over 52 million prescriptions issued annually.^[9]^ In the United Kingdom, omeprazole ranked third in 2023, closely followed by lansoprazole in fourth place, with both exceeding 34 million prescriptions.^[10]^ Although PPIs are generally considered safe within their therapeutic range, various adverse risks have been identified, including chronic kidney disease, dementia, myocardial infarction, and infections.^[11]^ Growing concerns regarding bone health have drawn attention to the potential effects of PPI use on bone integrity. Several studies have reported a possible association between PPI use and an elevated risk of osteoporosis and fractures, but the evidence remains inconclusive. While some observational studies have indicated a higher incidence of fractures or osteoporosis among PPI users,^[12–17]^ others have found no significant relationship between PPI use and bone-related health outcomes such as osteoporosis and fractures.^[18,19]^ A recent case-control study observed a reduction in bone mineral density (BMD) among long-term PPI users, though without a corresponding increase in fracture risk.^[20]^ It is crucial to recognize that many of these studies are observational, rendering them prone to biases such as reverse causation and confounding factors, which could affect the robustness of their conclusions.

Mendelian randomization (MR) analysis is an epidemiological approach rooted in Mendel’s laws of inheritance, gaining considerable attention for its ability to infer causal associations between risk factors and disease outcomes.^[21]^ This method leverages single nucleotide polymorphisms (SNPs) as instrumental variables (IVs) for exposure factors, enabling researchers to assess causality between exposures and outcomes.^[22]^ Given that specific SNP alleles are randomly assigned at conception, genetic variations are generally immune to confounding by external influences.^[23]^ In addition, since genetic variants are established prior to disease onset, MR analysis substantially mitigates the possibility of reverse causation.^[24]^ Consequently, this method is particularly effective for establishing causal relationships, bolstering the reliability and robustness of study findings.

A substantial body of MR studies has explored the causal factors underlying osteoporosis and fracture risk, investigating a range of variables, including socioeconomic status,^[25]^ type I diabetes mellitus,^[26]^ circulating inflammatory proteins,^[27]^ and nonalcoholic fatty liver disease.^[28]^ However, to date, no MR studies have examined the potential link between PPI use and the risk of osteoporosis or fractures. To address this research gap, the present study aims to elucidate the causal relationship between PPI use and the risk of osteoporosis and fractures through a two-sample MR analysis.

## 2. Methods

### 2.1. Study design

**Figure 1.**
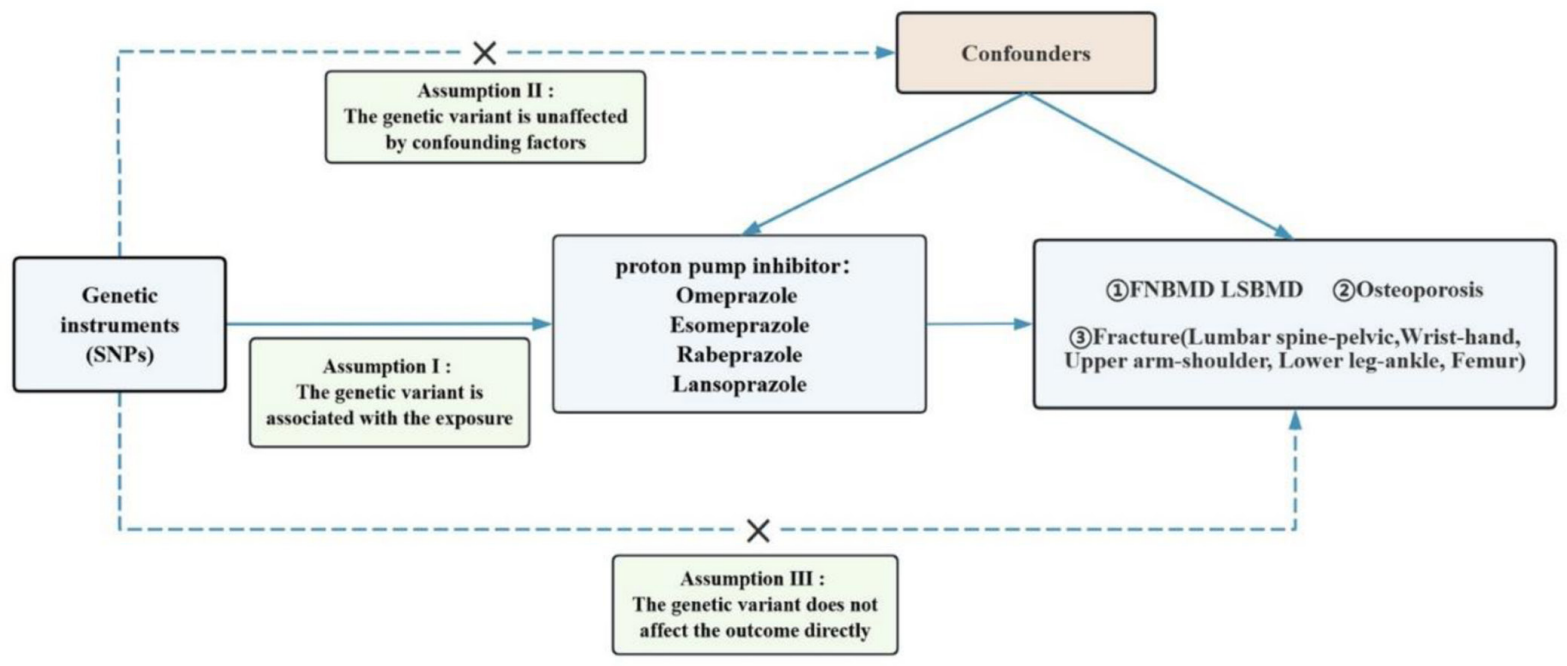
Design of the two-sample Mendelian randomization study. The 3 core assumptions are as follows: (I) relevance assumption; (II) independence assumption; and (III) exclusion restriction. The bold line represents a direct effect, and the dotted line represents an indirect effect. FNBMD = femoral neck bone mineral density, LSBMD = lumbar spine bone mineral density, SNP = single nucleotide polymorphism.

Figure 1 provides a comprehensive overview of our study design. We selected 4 PPIs (omeprazole, esomeprazole, lansoprazole, and rabeprazole) (N = 456,276), along with the 2 most representative BMDs (femoral neck BMD [FNBMD; N = 32,735] and lumbar spine BMD [N = 28,498]). Fracture data were analyzed at 5 anatomical sites: upper arm-shoulder(N = 389,773), wrist-hand (N = 379,425), lumbar spine-pelvic (N = 404,888), femur (N = 403,706), and lower leg-ankle (N = 374,644). Our MR analysis employed a unidirectional two-sample approach, adhering to 3 core assumptions^[29]^: the selected genetic variants needed to be strongly associated with the exposure; the genetic variants must be independent of confounding variables such as body mass index (BMI), smoking, and alcohol consumption; and the effect of the genetic variants on outcomes must be mediated exclusively through the exposure pathway. All MR analyses utilized publicly available summary statistics, eliminating the requirement for additional ethical approval or informed consent.

### 2.2. Data sources

The PPI data were derived from a genome-wide association study (GWAS) published in 2021, encompassing 456,276 participants of European ancestry.^[30]^ Additionally, GWAS summary statistics for osteoporosis (N = 399,054) and fractures at various anatomical sites were extracted from the recently released FinnGen dataset (accessible at https://r9.finngen.fi/). For BMD measurements (unit: g/cm^2^), data were sourced from the Genetic Factors for Osteoporosis Consortium (http://www.gefos.org/). Notably, all GWAS datasets were devoid of gender heterogeneity. The GWAS databases are publicly accessible and do not require permission for their use. Table 1 provides a detailed overview of these data sources, including the year of publication, consortium, ancestry, and sample size.

**Table 1:** Detailed GWAS data on exposure and outcome.

Phenotype	Year of publication	Consortium	Ancestry	Sample size	Number of case group	Number of control group
Exposure						
Omeprazole	2021	UK Biobank	European	456,276	26,869	429,407
Esomeprazole	2021	UK Biobank	European	456,276	1520	454,756
Rabeprazole	2021	UK Biobank	European	456,276	824	455,035
Lansoprazole	2021	UK Biobank	European	456,276	16,241	440,035
Outcome						
FNBMD	2015	GEFOS	European	32,735	NA	NA
LSBMD	2015	GEFOS	European	28,498	NA	NA
Osteoporosis	2023	FinnGen	European	399,054	8017	391,037
Upper arm-shoulder fracture	2023	FinnGen	European	389,773	12,920	376,853
Wrist-hand fracture	2023	FinnGen	European	379,425	12,701	366,724
Lumbar spine-pelvis fracture	2023	FinnGen	European	404,888	6831	398,057
Femur fracture	2023	FinnGen	European	403,706	9489	394,217
Lower leg-ankle fracture	2023	FinnGen	European	374,644	22,027	352,617

FNBMD = femoral neck bone mineral density, GEFOS = Genetic Factors for Osteoporosis Consortium, GWAS = genome-wide association study, LSBMD = lumbar spine bone mineral density.

### 2.3. Selection of instrumental variants

To ensure compliance with the assumptions of the MR analysis, we extracted SNPs strongly associated with the exposure, applying a genome-wide significance threshold of *P* < 5 × 10^−6^. A linkage disequilibrium test (*r*^2^ = 0.001, kb = 10,000) was then conducted on these SNPs to confirm their independence.^[31]^ The strength of the IVs was evaluated by calculating the *F*-statistic: *F* = *R*^2^(N − 2)/(1 − *R*^2^).^[32]^ SNPs with *F*-statistics below 10 were excluded to minimize bias from weak IVs.^[33]^ Additionally, the LDtrait Tool (National Cancer Institute, National Institutes of Health, https://ldlink.nih.gov/?tab=ldtrait) was used to filter out SNPs linked to potential confounders such as BMI, diabetes, smoking, alcohol consumption, and educational attainment, reducing the impact of horizontal pleiotropy. The remaining SNPs associated with PPI use were retained as IVs in the analysis. Detailed information on the PPI-associated SNPs can be found in Tables S1 to S4, Supplemental Digital Content 1.

### 2.4. MR analyses

To investigate the causal relationship between PPIs and the risk of osteoporosis and fracture, we utilized multiple MR analytical methods. The primary analysis was conducted using the inverse-variance weighting (IVW) approach, which calculates the Wald ratio-weighted mean across SNPs based on their exposure beta coefficients. This method provides a consistent estimate of the causal effect of the exposure on the outcomes, provided that each genetic variable meets the IV criteria.^[34]^ A fixed-effects IVW model was employed when no significant heterogeneity was detected, whereas a random-effects IVW model was applied in the presence of significant heterogeneity (*P* < .05).^[35]^ In addition, we implemented MR-Egger regression, which is particularly effective in testing the hypothesis of null causality and providing consistent causal assessments. This approach remains reliable even when all SNPs exhibit pleiotropy, as long as these pleiotropic effects are independent of their associations with the exposure.^[36]^ The weighted median method was also applied, offering robust causal estimates even if up to 50% of the analyzed data originates from invalid IVs.^[37]^ Finally, the Mendelian Randomization Pleiotropy Residual Sum and Outlier (MR-PRESSO) method was utilized to enhance result reliability by detecting and correcting for horizontal pleiotropy through the identification and exclusion of outliers.^[38]^

### 2.5. Sensitivity analysis

To ensure the reliability and robustness of our findings, we performed sensitivity analyses, including Cochran’s *Q* test, the MR-Egger intercept test, and leave-one-out (LOO) analysis. Cochran’s *Q* test was used to assess potential heterogeneity, examining whether variations in the IVs resulted in differential outcomes.^[39]^ A *P*-value of <.05 in this test indicated significant heterogeneity. The MR-Egger intercept test was employed to identify horizontal pleiotropy, where IVs potentially influence outcomes through pathways independent of the exposure. A *P*-value below .05 indicated the presence of horizontal pleiotropy.^[40]^ Subsequently, we conducted LOO analysis, which systematically excluded each SNP in turn and reestimated the effect estimates. Consistent results across the remaining SNPs would corroborate the robustness of the MR findings, confirming that no single SNP disproportionately influenced the effect estimates.

### 2.6. Statistical analysis

Given the multiple tests conducted in this study, we applied the Bonferroni correction to adjust for multiple comparisons, setting the threshold for statistical significance at a *P*-value of <.00156 (0.05/32). All analyses were executed using the *TwoSampleMR* package in R software (version 4.4.0; R Foundation for Statistical Computing).

## 3. Results

Through a comprehensive screening of GWAS data pertinent to PPIs, we identified 11 SNPs as IVs for omeprazole, 12 for esomeprazole, 9 for rabeprazole, and 17 for lansoprazole (Tables S1–S4, Supplemental Digital Content 1). Notably, the *F*-statistics for all variables exceeded 20, ensuring robust instrument validity. Furthermore, the MR-PRESSO method detected no evidence of outliers, reinforcing the reliability of the results.

Sensitivity analyses further corroborated the reliability and robustness of the findings presented in this study. No heterogeneity was observed across all analyses, justifying the application of the fixed-effects IVW method for the MR analyses. Additionally, the MR-Egger intercept test did not indicate any evidence of horizontal pleiotropy (Tables 2 and 3). Finally, the LOO analysis demonstrated that the majority of the MR estimates remained stable, with no single SNP disproportionately influencing the results, reinforcing the consistency of our findings (Figs. S1–S4, Supplemental Digital Content 5).

**Table 2:** Mendelian randomization results of the causal effect of PPIs on BMD and osteoporosis.

Exposures	Outcomes	No. of SNPs	Methods	β/OR (95% CI)	*P*	Heterogeneity test		Pleiotropy test
						Cochran’s *Q*	*P* *	*P* Intercept
Omeprazole	FNBMD	11	IVW	−0.015 (−0.121 to 0.090)	.776	3.54	.966	.594
			WM	−0.004 (−0.137 to 0.130)	.959			
			MR-Egger	0.063 (−0.234 to 0.360)	.687			
			MR-PRESSO	−0.015 (−0.078 to 0.048)	.644			
Omeprazole	LSBMD	11	IVW	−0.075 (−0.215 to 0.065)	.292	13.08	.219	.307
			WM	0.019 (−0.158 to 0.197)	.832			
			MR-Egger	0.344 (0.001–0.688)	.175			
			MR-PRESSO	−0.085 (−0.232 to 0.062)	.280			
Omeprazole	Osteoporosis	11	IVW	0.965 (0.774–1.203)	.752	6.14	.803	.435
			WM	0.980 (0.732–1.313)	.892			
			MR-Egger	1.223 (0.665–2.247)	.533			
			MR-PRESSO	0.941 (0.790–1.120)	.505			
Esomeprazole	FNBMD	10	IVW	0.001 (−0.026 to 0.028)	.934	12.80	.235	.731
			WM	−0.008 (−0.039 to 0.024)	.620			
			MR-Egger	−0.006 (−0.056 to 0.044)	.809			
			MR-PRESSO	0.001 (−0.026 to 0.028)	.936			
Esomeprazole	LSBMD	10	IVW	0.007 (−0.021 to 0.034)	.623	4.62	.915	.587
			WM	0.018 (−0.017 to 0.052)	.323			
			MR-Egger	0.019 (−0.031 to 0.068)	.478			
			MR-PRESSO	0.007 (−0.012 to 0.026)	.486			
Esomeprazole	Osteoporosis	11	IVW	0.974 (0.929–1.022)	.286	4.34	.959	.719
			WM	0.973 (0.915–1.035)	.387			
			MR-Egger	0.963 (0.892–1.041)	.366			
			MR-PRESSO	0.974 (0.946–1.004)	.117			
Rabeprazole	FNBMD	9	IVW	0.011 (−0.012 to 0.034)	.358	10.31	.244	.530
			WM	0.011 (−0.016 to 0.039)	.416			
			MR-Egger	−0.002 (−0.048 to 0.044)	.926			
			MR-PRESSO	0.011 (−0.012 to 0.034)	.385			
Rabeprazole	LSBMD	9	IVW	−0.009 (−0.035 to 0.017)	.487	9.39	.311	.823
			WM	0.004 (−0.030 to 0.0380)	.809			
			MR-Egger	−0.004 (−0.056 to 0.049)	.889			
			MR-PRESSO	−0.009 (−0.035 to 0.017)	.507			
Rabeprazole	Osteoporosis	7	IVW	0.979 (0.943–1.017)	.273	4.31	.634	.226
			WM	0.992 (0.945–1.041)	.732			
			MR-Egger	1.015 (0.953–1.081)	.666			
			MR-PRESSO	0.979 (0.949–1.011)	.244			
Lansoprazole	FNBMD	15	IVW	0.137 (0.063–0.210)	2.73E−04	15.38	.353	.334
			WM	0.140 (0.042–0.238)	4.90E−03			
			MR-Egger	0.212 (0.047–0.377)	2.57E−02			
			MR-PRESSO	0.137 (0.063–0.210)	2.68E−03			
Lansoprazole	LSBMD	15	IVW	0.064 (−0.034 to 0.163)	.202	20.84	.106	.474
			WM	0.106 (−0.002 to 0.213)	.053			
			MR-Egger	0.141 (−0.086 to 0.367)	.245			
			MR-PRESSO	0.064 (−0.034 to 0.163)	.223			
Lansoprazole	Osteoporosis	15	IVW	1.089 (0.944–1.257)	.242	10.36	.735	.814
			WM	1.099 (0.898–1.345)	.357			
			MR-Egger	1.058 (0.803–1.394)	.693			
			MR-PRESSO	1.089 (0.963–1.232)	.195			

CI = confidence interval, FNBMD = femoral neck bone mineral density, IVW = inverse-variance weighting, LSBMD = lumbar spine bone mineral density, MR-PRESSO = Mendelian Randomization Pleiotropy Residual Sum and Outlier, OR = odds ratio, PPI = proton pump inhibitor, SNPs = single nucleotide polymorphisms, WM = weighted median.

* *P* represents heterogeneity.

**Table 3:** Mendelian randomization results of the causal effect of PPIs on fractures.

PPIs	Fracture	No. of SNPs	Methods	β (95% CI)	*P*	Heterogeneity test		Pleiotropy test
						Cochran’s *Q*	*P* *	*P* Intercept
Omeprazole	Upper arm-shoulder	11	IVW	0.903 (0.712–1.146)	.313	14.39	.156	.323
			WM	0.670 (0.381–1.180)	.402			
			MR-Egger	0.899 (0.731–1.106)	.199			
			MR-PRESSO	1.052 (0.884–1.253)	.337			
Omeprazole	Wrist-hand	11	IVW	1.176 (0.919–1.506)	.568	9.60	.476	.147
			WM	1.513 (0.935–2.448)	.198			
			MR-Egger	1.052 (0.887–1.248)	.126			
			MR-PRESSO	1.104 (0.871–1.397)	.573			
Omeprazole	Lumbar spine-pelvic	11	IVW	1.205 (0.881–1.649)	.414	6.70	.754	.511
			WM	1.363 (0.711–2.613)	.242			
			MR-Egger	1.104 (0.910–1.339)	.375			
			MR-PRESSO	1.078 (0.861–1.350)	.341			
Omeprazole	Femur	11	IVW	1.169 (0.866–1.577)	.512	12.08	.280	.650
			WM	1.246 (0.652–2.380)	.307			
			MR-Egger	1.078 (0.861–1.350)	.522			
			MR-PRESSO	1.061 (0.912–1.236)	.526			
Omeprazole	Lower leg-ankle	11	IVW	1.113 (0.919–1.348)	.443	12.94	.227	.598
			WM	0.948 (0.614–1.465)	.275			
			MR-Egger	1.061 (0.912–1.236)	.816			
			MR-PRESSO	0.903 (0.712–1.146)	.460			
Esomeprazole	Upper arm-shoulder	11	IVW	1.023 (0.986–1.062)	.228	4.85	.938	.673
			WM	1.024 (0.975–1.074)	.346			
			MR-Egger	1.034 (0.973–1.098)	.304			
			MR-PRESSO	1.023 (0.998–1.049)	.097			
Esomeprazole	Wrist-hand	11	IVW	1.008 (0.971–1.047)	.666	6.70	.823	.642
			WM	1.017 (0.965–1.072)	.529			
			MR-Egger	1.020 (0.960–1.084)	.535			
			MR-PRESSO	1.008 (0.979–1.038)	.592			
Esomeprazole	Lumbar spine-pelvic	11	IVW	1.015 (0.964–1.068)	.573			
			WM	1.019 (0.951–1.093)	.584	5.61	.898	.716
			MR-Egger	1.002 (0.923–1.089)	.961			
			MR-PRESSO	1.015 (0.979–1.052)	.447			
Esomeprazole	Femur	11	IVW	1.049 (1.004–1.096)	.031	5.04	.888	.943
			WM	1.035 (0.976–1.098)	.248			
			MR-Egger	1.049 (1.004–1.125)	.230			
			MR-PRESSO	1.049 (1.019–1.081)	.009			
Esomeprazole	Lower leg-ankle	11	IVW	1.027 (0.996–1.059)	.093	12.70	.313	.526
			WM	1.028 (0.985–1.072)	.209			
			MR-Egger	1.013 (0.962–1.067)	.640			
			MR-PRESSO	1.027 (0.996–1.059)	.121			
Rabeprazole	Upper arm-shoulder	7	IVW	0.976 (0.947–1.005)	.101	3.98	.679	.474
			WM	0.980 (0.941–1.021)	.335			
			MR-Egger	0.960 (0.914–1.009)	.171			
			MR-PRESSO	0.976 (0.953–0.999)	.090			
Rabeprazole	Wrist-hand	7	IVW	0.991 (0.956–1.028)	.624	8.90	.179	.098
			WM	0.990 (0.952–1.030)	.634			
			MR-Egger	0.950 (0.904–0.999)	.103			
			MR-PRESSO	0.991 (0.956–1.028)	.642			
Rabeprazole	Lumbar spine-pelvic	7	IVW	1.009 (0.970–1.051)	.647	5.38	.496	.631
			WM	0.991 (0.941–1.044)	.740			
			MR-Egger	1.024 (0.956–1.097)	.526			
			MR-PRESSO	1.009 (0.972–1.049)	.646			
Rabeprazole	Femur	7	IVW	0.976 (0.942–1.011)	.170	2.02	.918	.803
			WM	0.982 (0.938–1.028)	.435			
			MR-Egger	0.970 (0.914–1.029)	.354			
			MR-PRESSO	0.976 (0.956–0.996)	.056			
Rabeprazole	Lower leg-ankle	7	IVW	1.002 (0.968–1.038)	.893	14.23	.027	.889
			WM	1.000 (0.970–1.032)	.978			
			MR-Egger	1.006 (0.943–1.073)	.856			
			MR-PRESSO	1.002 (0.968–1.038)	.898			
Lansoprazole	Upper arm-shoulder	15	IVW	1.030 (0.922–1.151)	.602	12.39	.575	.253
			WM	1.041 (0.890–1.218)	.614			
			MR-Egger	0.922 (0.746–1.141)	.469			
			MR-PRESSO	1.030 (0.928–1.144)	.588			
Lansoprazole	Wrist-hand	15	IVW	1.054 (0.936–1.187)	.385	15.57	.340	.808
			WM	1.023 (0.865–1.210)	.789			
			MR-Egger	1.081 (0.855–1.366)	.526			
			MR-PRESSO	1.054 (0.936–1.187)	.399			
Lansoprazole	Lumbar spine-pelvic	15	IVW	1.009 (0.866–1.176)	.906	11.74	.628	.853
			WM	1.097 (0.890–1.353)	.384			
			MR-Egger	1.034 (0.772–1.385)	.827			
			MR-PRESSO	1.009 (0.878–1.161)	.899			
Lansoprazole	Femur	15	IVW	0.999 (0.876–1.140)	.991	11.01	.685	.618
			WM	0.997 (0.830–1.199)	.975			
			MR-Egger	1.057 (0.821–1.362)	.674			
			MR-PRESSO	0.999 (0.889–1.123)	.990			
Lansoprazole	Lower leg-ankle	15	IVW	0.961 (0.882–1.048)	.371	7.67	.906	.252
			WM	0.933 (0.828–1.051)	.252			
			MR-Egger	0.882 (0.748–1.041)	.160			
			MR-PRESSO	0.961 (0.902–1.025)	.247			

CI = confidence interval, IVW = inverse-variance weighting, MR-PRESSO = Mendelian Randomization Pleiotropy Residual Sum and Outlier, PPI = proton pump inhibitor, SNPs = single nucleotide polymorphisms, WM = weighted median.

* *P* represents heterogeneity.

### 3.1. The causal effect of PPIs on BMD and osteoporosis

**Figure 2.**
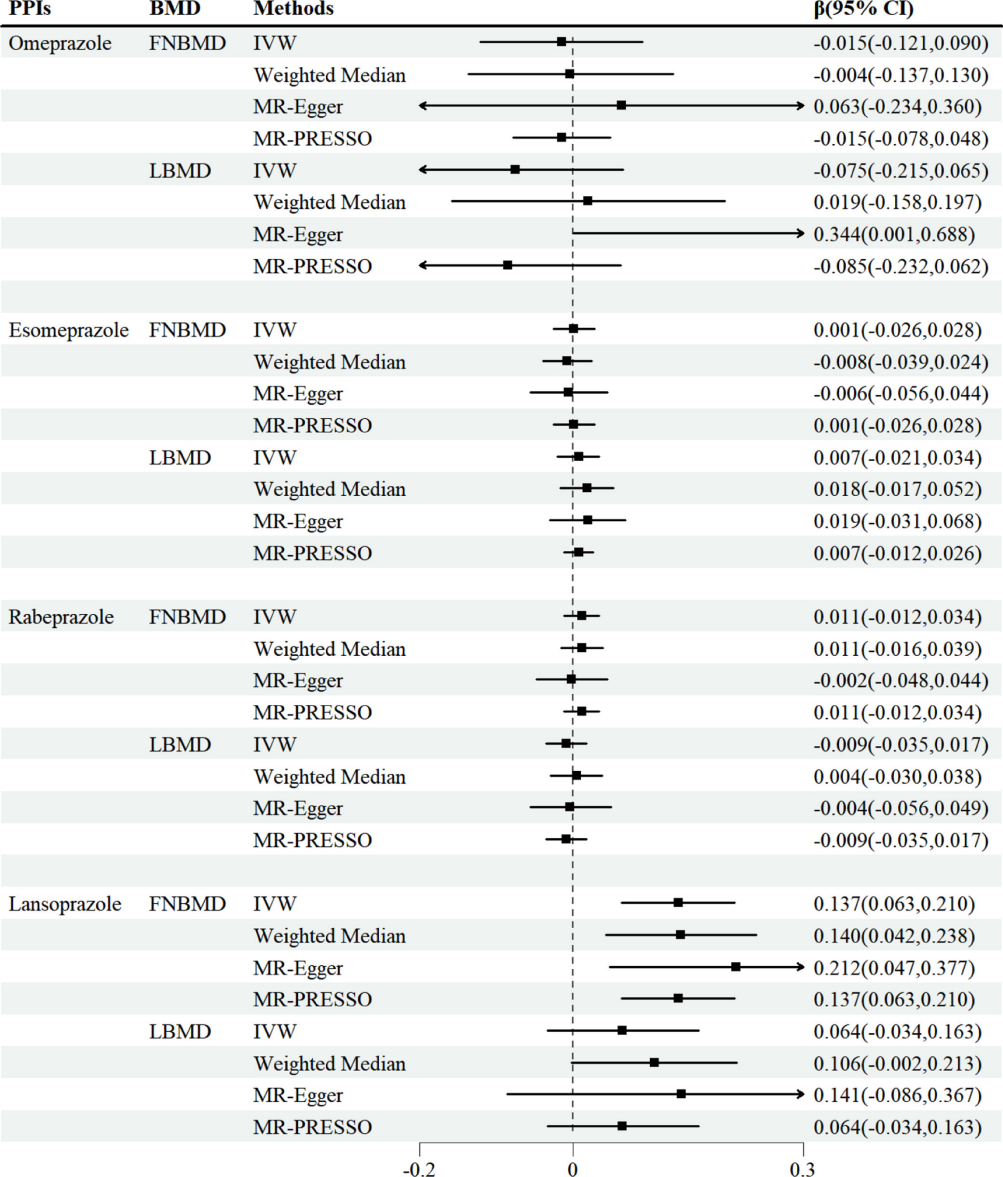
Forest plot of the causal effects of PPIs on BMD. FNBMD = femoral neck bone mineral density, CI = confidence interval, IVW = inverse-variance weighting, LSBMD = lumbar spine bone mineral density, PPIs = proton pump inhibitors.

**Figure 3.**
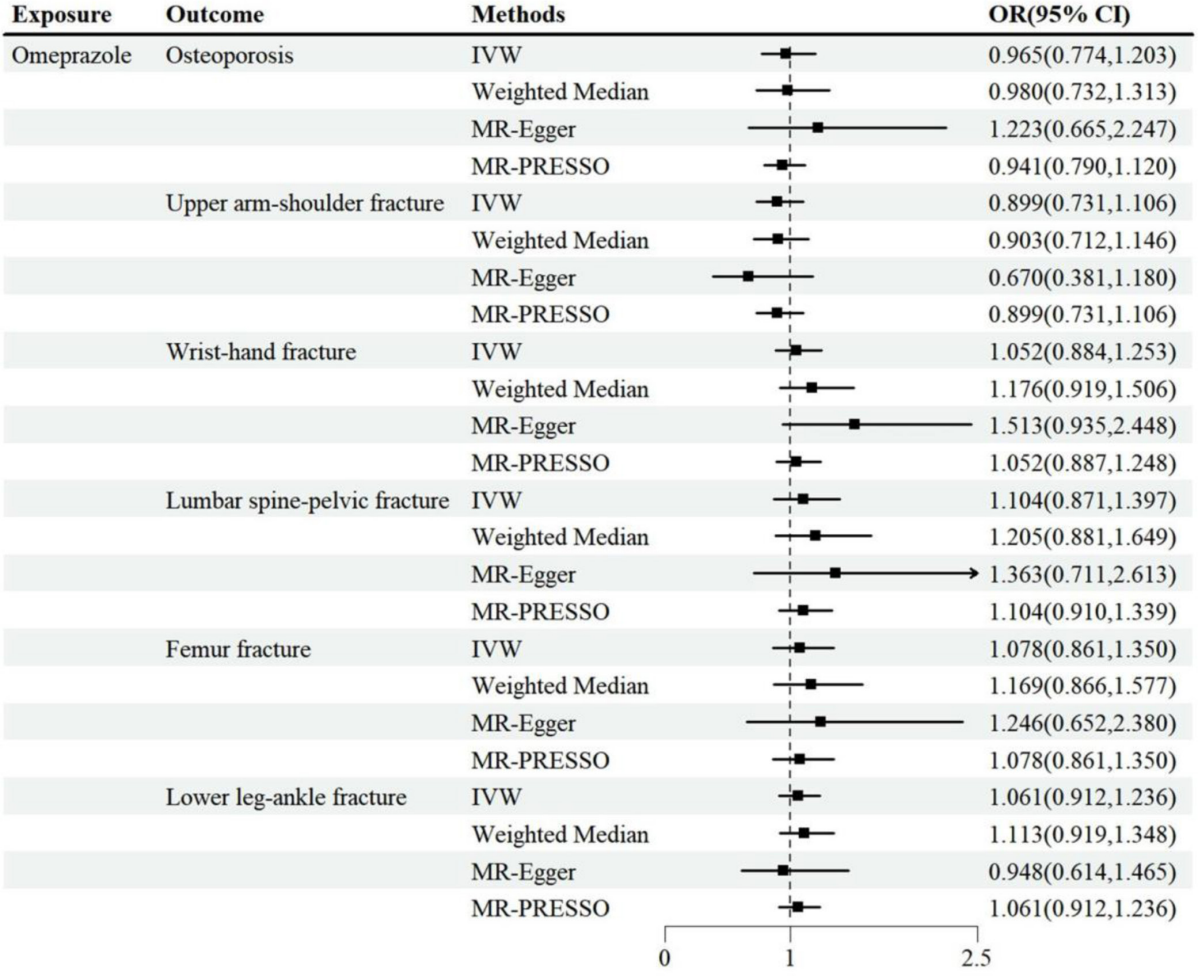
Forest plot of the causal effects of omeprazole on osteoporosis and fractures. CI = confidence interval, IVW = inverse-variance weighting, OR = odds ratio.

**Figure 4.**
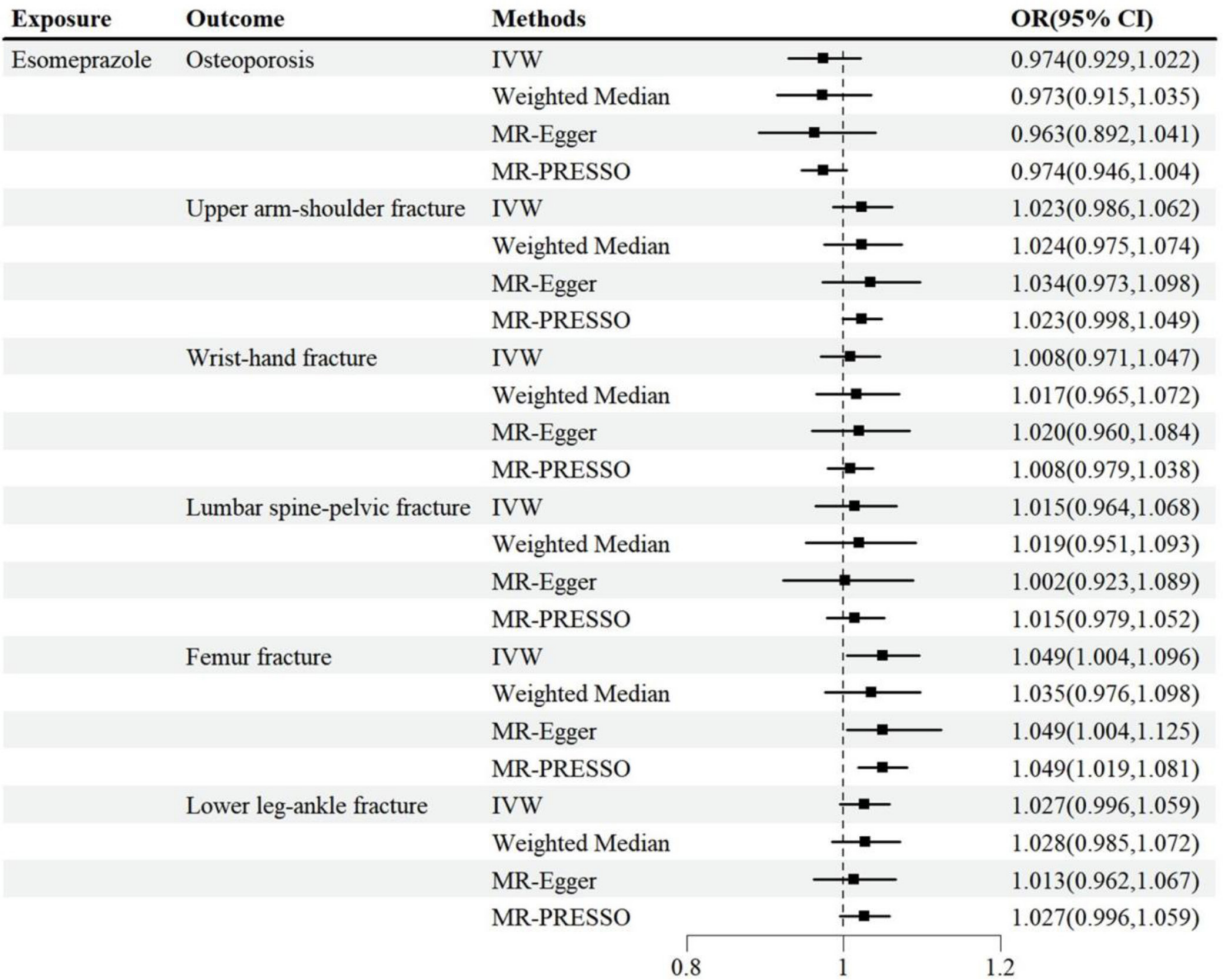
Forest plot of the causal effects of esomeprazole on osteoporosis and fractures. CI = confidence interval, IVW = inverse-variance weighting, OR = odds ratio.

**Figure 5.**
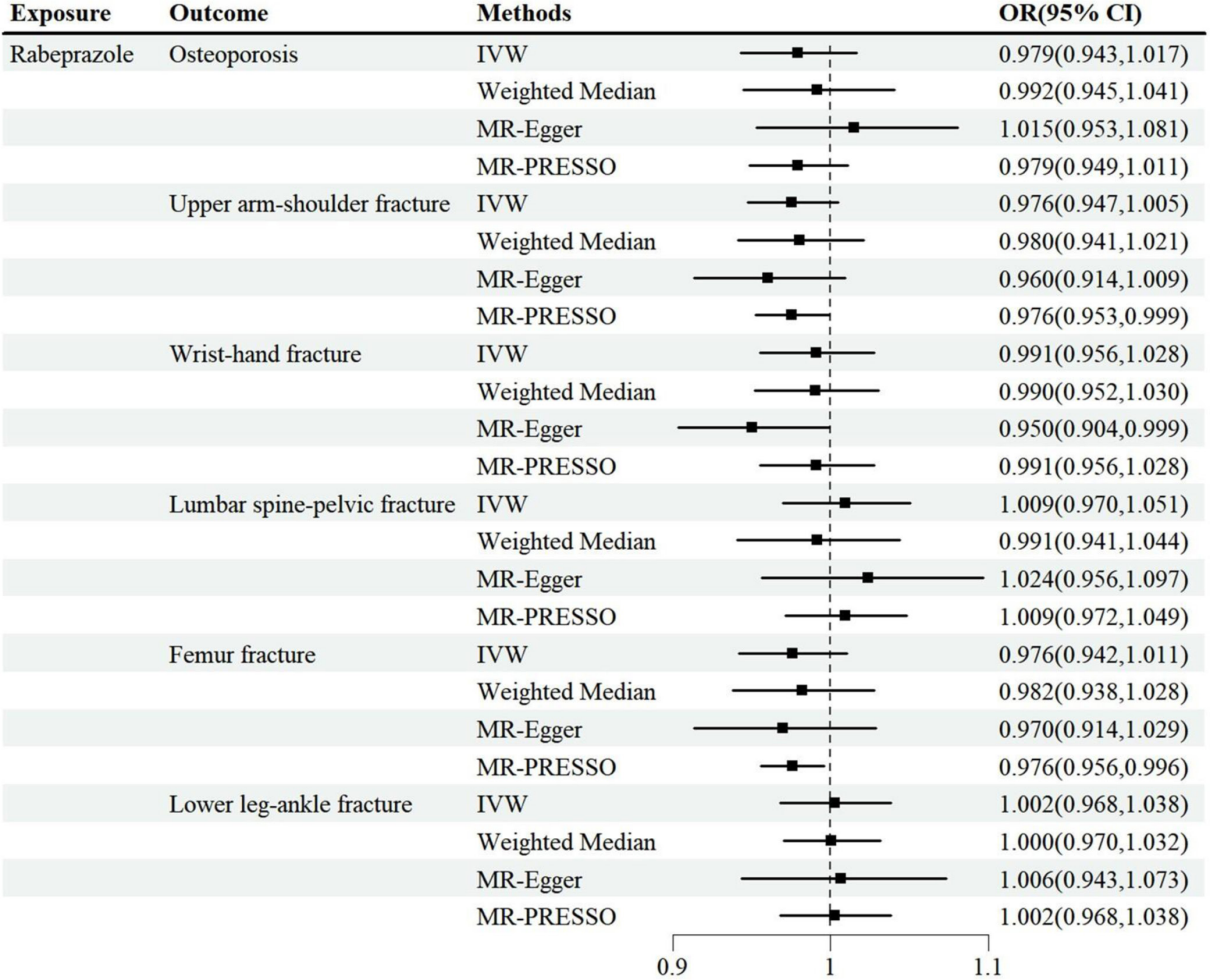
Forest plot of the causal effects of rabeprazole on osteoporosis and fractures. CI = confidence interval, IVW = inverse-variance weighting, OR = odds ratio.

**Figure 6.**
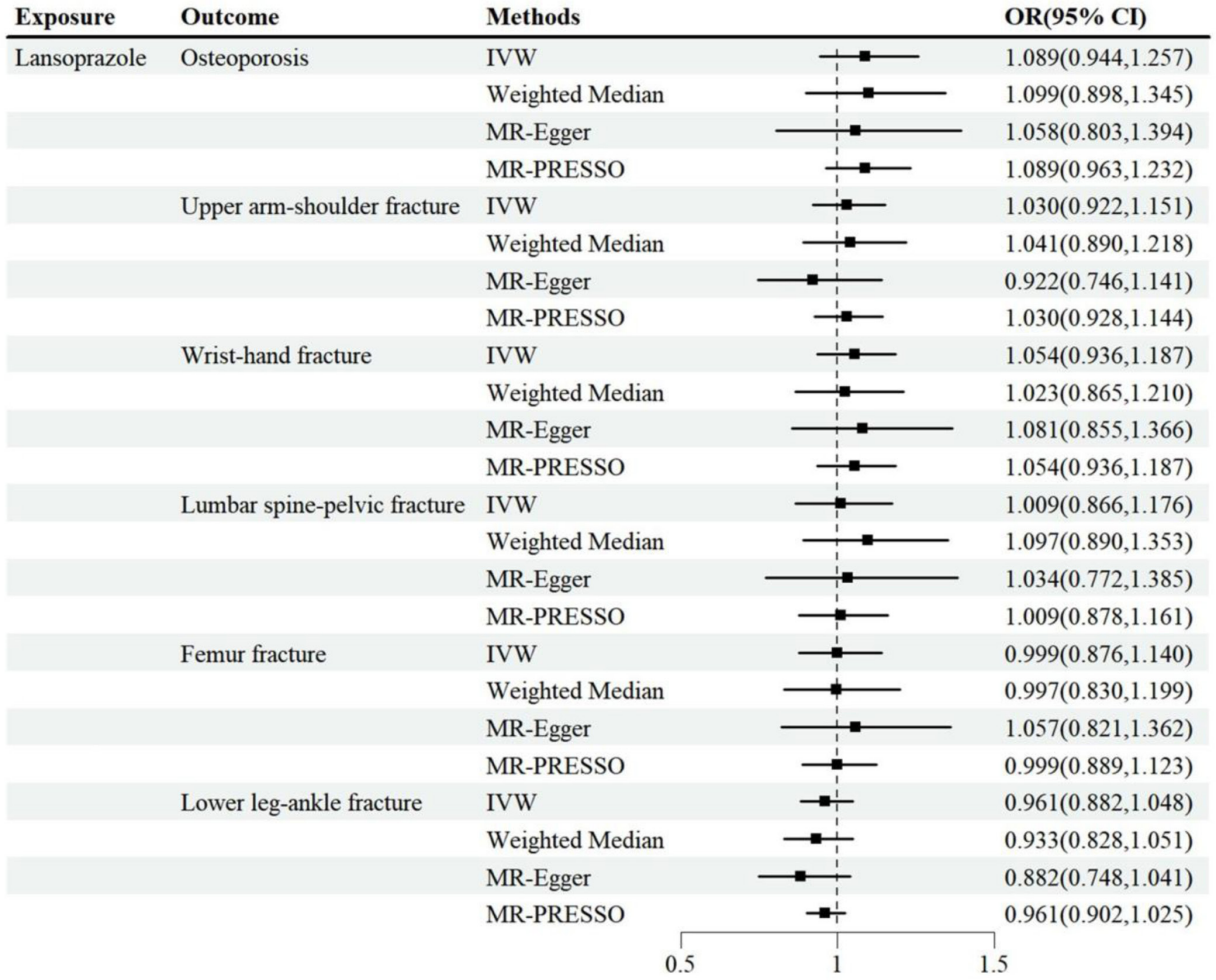
Forest plot of the causal effects of lansoprazole on osteoporosis and fractures. CI = confidence interval, IVW = inverse-variance weighting, OR = odds ratio.

MR estimates assessing the causal effect of PPIs on BMD and osteoporosis revealed a significant positive relationship between lansoprazole and FNBMD (IVW: β = 0.137, 95% confidence interval [CI]: 0.063–0.210, *P* = 2.73E−04), which persisted following Bonferroni correction. This association was supported by alternative MR approaches, which yielded consistent results (WM: β = 0.140, 95% CI: 0.042–0.238, *P* = 4.90E−03; MR-Egger: β = 0.212, 95% CI: 0.047–0.377, *P* = 2.57E−02; MR-PRESSO: β = 0.137, 95% CI: 0.063–0.210, *P* = 2.68E−03). No causal association was identified between lansoprazole and lumbar spine BMD or osteoporosis. Similarly, no causal effect on BMD or osteoporosis was detected for the other PPIs, including omeprazole, esomeprazole, and rabeprazole (Tables 2 and 3; Figs. 2–6).

### 3.2. The causal effect of PPIs on fractures

In this MR analysis examining the causal effect of PPIs on fracture risk, an initial positive causal association was found between esomeprazole and femur fracture (IVW: odds ratio = 1.049, 95% CI: 1.004–1.096, *P* = .031). However, this association lost statistical significance following Bonferroni correction, indicating that esomeprazole may exert only a marginal causal impact on femur fracture risk. Additionally, esomeprazole was not associated with fracture risk at other anatomical sites. Likewise, no causal associations were identified between other PPIs, including omeprazole, rabeprazole, and lansoprazole, and fracture risk at various sites (Tables 2 and 3; Figs. 3–6).

## 4. Discussion

This MR study explored the potential causal link between PPI use and bone health outcomes, including BMD at the femoral neck and lumbar spine, osteoporosis, and fracture risk across various skeletal sites. Our findings indicate no overall association between PPI use and adverse bone health effects. Specifically, the MR analysis demonstrated that neither omeprazole nor rabeprazole exerted any discernible effects on BMD, osteoporosis, or fracture risk. While lansoprazole was not significantly associated with osteoporosis or fracture risk, it was notably linked to a positive effect on FNBMD, suggesting a potential protective influence on bone health. Conversely, esomeprazole exhibited only a marginal association with an increased risk of femoral fracture, with no significant associations with other bone health outcomes. These results provide valuable insights into the ongoing debate regarding the safety of PPI use, particularly among populations vulnerable to bone loss and fractures.

While numerous prior studies have indicated that PPI usage may result in reduced BMD and an increased risk of osteoporosis and fractures, the overall evidence remains inconsistent. An animal study demonstrated that high-dose lansoprazole treatment led to diminished BMD and degradation of bone microarchitecture.^[41]^ Similarly, observational studies conducted in Taiwan,^[42]^ Iran,^[14]^ Korea,^[17]^ and Germany^[43]^ reported a heightened risk of osteoporosis among individuals using PPIs. Further investigations from Canada^[12]^ and the United States^[44]^ have established a significant correlation between PPI use and an increased incidence of fractures. However, other studies have failed to establish a substantial link between PPI use and accelerated BMD loss,^[45–47]^ with several prospective investigations finding no association between long-term PPI use and reductions in bone strength or impairments in bone microarchitecture.^[19,48,49]^ Additionally, some research has suggested that PPI use does not correlate with an elevated risk of fractures.^[18,50,51]^ Our findings align with this latter perspective, indicating no causal association between PPI use and alterations in BMD, osteoporosis development, or fracture risk.

PPIs are widely prescribed as therapeutic agents for gastric acid-related disorders, functioning by inhibiting the H^+^/K^+^-ATPase enzyme in the gastric epithelium, thereby reducing gastric acid secretion.^[52]^ Previous research has suggested that prolonged PPI use leads to diminished gastric acid secretion, potentially interfering with calcium absorption. It has been posited that gastric acid plays a pivotal role in the solubilization and absorption of calcium, as calcium in its nonionic state requires an acidic environment for effective dissolution prior to absorption.^[53]^ Insufficient calcium absorption can disrupt calcium homeostasis, adversely affecting bone mineralization and potentially contributing to decreased BMD.^[12]^ However, other evidence suggests that a reduction in gastric acid does not significantly impact calcium absorption. Although gastric acid can facilitate the dissolution of calcium under certain circumstances, effective pathways exist for calcium absorption in the gastrointestinal tract, particularly for dietary sources. Calcium derived from food typically exists in a chelated form and does not wholly rely on an acidic gastric environment for absorption.^[54]^ Consequently, under typical dietary conditions, the influence of PPIs on calcium absorption appears to be modest and may not be sufficient to substantially reduce BMD or increase fracture risk.^[55]^

Moreover, bone metabolism is regulated by a complex interplay of factors, indicating that the risk of osteoporosis and fractures is not determined solely by calcium absorption or gastric acid secretion.^[52]^ Other determinants, including genetic predisposition, hormonal levels, and BMI, may account for a substantial portion of this risk.^[56]^ The elevated fracture risk observed among long-term PPI users may, therefore, be more attributable to underlying factors than to the direct effects of PPIs.^[45]^ Supporting this perspective, a study found no statistically significant association between PPI use and fracture rates after adjusting for multiple confounding factors.^[57]^ Researchers posited that discrepancies in findings could be attributed to variations in study design, sample size, and the control of confounding factors, rather than evidence of a direct detrimental effect of PPIs on bone health.^[58]^ Given the limited impact of PPIs on calcium absorption and the multifactorial nature of bone health, PPI use may not represent a major determinant of osteoporosis and fracture risk.

Interestingly, our study revealed a positive association between lansoprazole use and FNBMD. This novel finding suggests that specific PPIs may exert differential effects on bone health. Previous research has reported mixed results regarding the effects of various PPIs on BMD. For instance, Bahtiri et al found that esomeprazole was significantly associated with reduced BMD, whereas omeprazole demonstrated no effect.^[59]^ In contrast, Hansen et al reported that neither esomeprazole nor dexlansoprazole had a significant impact on bone homeostasis in postmenopausal women.^[60]^ The differential effects observed in our study may be related to the unique pharmacokinetic properties of lansoprazole, including its more rapid onset of action and shorter half-life, which may attenuate its influence on calcium absorption and bone metabolism compared with other PPIs.^[41]^

It is worth noting that most studies investigating the association between PPI use and adverse events have relied on observational analyses, which are inherently susceptible to confounding and reverse causality. Observational studies may yield spurious associations, as PPIs are often prescribed to individuals with multiple comorbidities, which themselves may act as confounders. In contrast, the application of MR in our study effectively mitigated these limitations by addressing confounding and reverse causality. Additionally, the data used were sourced exclusively from individuals of European ancestry, minimizing heterogeneity. The use of GWAS data from 2 independent samples further enhanced the statistical power of our analysis.

Despite its strengths, our study has several limitations. First, our study population was confined to individuals of European ancestry, potentially limiting the generalizability of our findings to other ethnic and demographic groups. Second, the absence of GWAS data stratified by sex or age prevented us from evaluating whether the associations between PPIs and bone health outcomes differ across sexes or age groups. Third, the exposure definitions were derived from UK Biobank GWAS data, in which each medication phenotype was coded as use versus nonuse of a specific drug within the full cohort. Consequently, the control group for a given PPI may have included individuals using other PPIs. This potential overlap could introduce exposure misclassification and attenuate the estimated associations, thereby biasing the results toward the null. Although this limitation is inherent to the use of publicly available summary-level data in a two-sample MR framework and does not violate the core MR assumptions, it may reduce the specificity of the estimated drug effects and should be considered when interpreting our findings. Finally, while our study focused on the causal effects of PPI use on osteoporosis and fracture risk, it did not explore the specific biological mechanisms underlying these associations. Future research is needed to elucidate these mechanisms and validate our findings in diverse populations.

## 5. Conclusion

In conclusion, our MR analysis found no evidence of a causal link between PPI use and adverse bone health outcomes, including BMD, osteoporosis, and fracture risk, with the exception of a positive association between lansoprazole use and FNBMD. Further research is essential to elucidate the biological mechanisms underlying these observations and to evaluate the safety profiles of specific PPIs across different demographic groups.

## Author contributions

**Data curation:** Ping Li, Ruiji Wu.

**Formal analysis:** Ping Li.

**Software:** Ping Li.

**Methodology:** Hangchu Shi.

**Writing – original draft:** Ping Li.

**Writing – review & editing:** Ping Li, Ruiji Wu, Hangchu Shi.
















