# Supplementary material for: Proton pump inhibitors use and the risk of osteoporosis and fractures: A two-sample Mendelian randomization study
Source: Medicine (Baltimore). 2026 Jul 24;105(30):e49964. doi: 10.1097/MD.0000000000049964 (PMC13406325; doi:10.1097/MD.0000000000049964)
Supplement: Supplementary file 1 [file medi-105-e49964-s001.docx]

Table S1 Characteristics of SNPs used as genetic instruments for omeprazole

| SNP | Position | EA | NEA | EAF | SNP-Exposure association | | | R^2 a^ | F-statistic ^b^ | Confounders ^c^ |
| --- | --- | --- | --- | --- | --- | --- | --- | --- | --- | --- |
|  |  |  |  |  | Beta | SE | P value |  |  |  |
| rs115207920 | 454778 | A | T | 0.039 | -0.108 | 0.024 | 4.58E-06 | 4.73E-05 | 21.01 |  |
| rs3817459 | 1265686 | A | G | 0.308 | 0.049 | 0.01 | 6.90E-07 | 5.65E-05 | 24.64 | Educational attainment |
| rs4676893 | 2257825 | A | T | 0.305 | 0.049 | 0.01 | 6.29E-07 | 5.59E-05 | 24.82 | Alcohol consumption |
| rs62282693 | 2581298 | T | A | 0.166 | -0.057 | 0.012 | 3.57E-06 | 4.80E-05 | 21.48 |  |
| rs13100451 | 2731565 | A | G | 0.183 | -0.056 | 0.012 | 2.17E-06 | 4.99E-05 | 22.44 | Type 2 diabetes |
| rs4623058 | 3610591 | T | A | 0.179 | -0.055 | 0.012 | 2.90E-06 | 4.82E-05 | 21.88 |  |
| rs647035 | 4601883 | A | G | 0.381 | 0.049 | 0.01 | 3.55E-07 | 6.12E-05 | 25.92 | Type 2 diabetes,BMI |
| rs6464618 | 5837392 | T | C | 0.409 | -0.043 | 0.009 | 3.23E-06 | 4.90E-05 | 21.68 |  |
| rs4240631 | 5985142 | G | A | 0.269 | 0.048 | 0.01 | 2.34E-06 | 4.93E-05 | 22.3 | Type 2 diabetes,BMI |
| rs76982774 | 6305577 | A | G | 0.015 | -0.173 | 0.037 | 3.25E-06 | 4.75E-05 | 21.66 |  |
| rs4422827 | 6897675 | T | A | 0.477 | 0.043 | 0.009 | 2.78E-06 | 4.94E-05 | 21.96 | Educational attainment |
| rs75504366 | 6968434 | G | C | 0.028 | 0.126 | 0.027 | 3.26E-06 | 4.76E-05 | 21.66 |  |
| rs11790051 | 7036710 | C | G | 0.244 | -0.051 | 0.01 | 9.70E-07 | 5.26E-05 | 23.99 | Smoking initiation |
| rs11040831 | 7725767 | A | G | 0.167 | 0.062 | 0.012 | 7.66E-07 | 5.70E-05 | 24.44 | BMI,Smoking initiation |
| rs17092943 | 8218211 | T | C | 0.212 | 0.051 | 0.011 | 4.10E-06 | 4.68E-05 | 21.22 | Educational attainment |
| rs79454971 | 8518467 | T | C | 0.041 | 0.105 | 0.023 | 3.64E-06 | 4.75E-05 | 21.45 |  |
| rs9598273 | 9026684 | G | A | 0.287 | 0.05 | 0.01 | 9.77E-07 | 5.61E-05 | 23.97 |  |
| rs79887313 | 10693197 | C | T | 0.042 | 0.105 | 0.023 | 3.20E-06 | 4.79E-05 | 21.7 | smoking initiation |
| rs28588631 | 10920393 | A | G | 0.214 | 0.05 | 0.011 | 4.95E-06 | 4.62E-05 | 20.86 | BMI |
| rs78484848 | 11079702 | T | C | 0.097 | 0.083 | 0.015 | 5.51E-08 | 6.49E-05 | 29.53 |  |
| rs7276225 | 11634159 | T | C | 0.298 | 0.049 | 0.01 | 1.09E-06 | 5.42E-05 | 23.77 |  |
| rs384262 | 11693232 | G | A | 0.412 | -0.045 | 0.009 | 7.76E-07 | 5.44E-05 | 24.42 |  |

Abbreviation: SNP, single nucleotide polymorphism; EA, Effect allele; NEA, Non-effect allele; EAF, effect allele frequency; SE, standard error; BMI, body mass index.

*^a^ R^2^* was calculated the following formula:(2×EAF×(1-EAF)×beta^2^)/[(2×EAF×(1-EAF)×beta^2^)+(2×EAF×(1-EAF)×N×SE^2^)],

where EAF is the effect allele frequency, beta is the estimated effect on urate. Ν is the sample size of the GWAS for the SNP-urate association and SE is the standard error of the estimated effect.

*^b^ F* statistic was calculated using the following formula: *R^2^*(N-2)/(1-*R^2^*), where *R^2^* is the proportion of variance in urate explained by each instrument and N is the sample size of the GWAS for the SNP-urate association.

^c^ SNPs associated with confounding factors were removed after searching LDlink.
