# Supplementary material for: Proton pump inhibitors use and the risk of osteoporosis and fractures: A two-sample Mendelian randomization study
Source: Medicine (Baltimore). 2026 Jul 24;105(30):e49964. doi: 10.1097/MD.0000000000049964 (PMC13406325; doi:10.1097/MD.0000000000049964)
Supplement: Supplementary file 2 [file medi-105-e49964-s002.docx]

Table S2 Characteristics of SNPs used as genetic instruments for esomeprazole

| SNP | Position | EA | NEA | EAF | SNP-Exposure association | | | R^2 a^ | F-statistic ^b^ | Confounders ^c^ |
| --- | --- | --- | --- | --- | --- | --- | --- | --- | --- | --- |
|  |  |  |  |  | Beta | SE | P value |  |  |  |
| rs12041365 | 728304 | A | G | 0.379 | -0.175 | 0.038 | 3.40E-06 | 4.77E-05 | 21.57 |  |
| rs55939202 | 1423235 | C | T | 0.418 | -0.174 | 0.037 | 2.92E-06 | 4.87E-05 | 21.87 |  |
| rs56070233 | 2116442 | T | C | 0.011 | 0.884 | 0.187 | 2.17E-06 | 4.92E-05 | 22.44 | Educational attainment |
| rs73095178 | 2262734 | G | A | 0.121 | -0.274 | 0.058 | 2.16E-06 | 5.20E-05 | 22.45 | Alcohol consumption |
| rs115067852 | 2363601 | G | A | 0.007 | 1.13 | 0.245 | 3.80E-06 | 4.75E-05 | 21.36 |  |
| rs76358556 | 2479667 | G | A | 0.204 | 0.221 | 0.045 | 1.19E-06 | 5.17E-05 | 23.6 |  |
| rs139754449 | 4089523 | C | G | 0.023 | 0.713 | 0.13 | 3.79E-08 | 6.66E-05 | 30.26 |  |
| rs3111476 | 5874958 | T | C | 0.064 | 0.351 | 0.075 | 3.17E-06 | 4.76E-05 | 21.71 |  |
| rs139294193 | 6174193 | G | C | 0.002 | 2.34 | 0.478 | 9.79E-07 | 5.28E-05 | 23.97 |  |
| rs4478540 | 6404521 | T | A | 0.039 | 0.479 | 0.097 | 8.65E-07 | 5.32E-05 | 24.21 |  |
| rs7820543 | 6463034 | C | A | 0.389 | -0.172 | 0.037 | 3.93E-06 | 4.67E-05 | 21.3 |  |
| rs117201520 | 8988596 | C | G | 0.012 | 0.923 | 0.186 | 6.55E-07 | 5.42E-05 | 24.74 |  |
| rs72671454 | 9305182 | A | G | 0.051 | 0.389 | 0.084 | 4.25E-06 | 4.64E-05 | 21.15 | Educational attainment |
| rs117656972 | 10068961 | T | C | 0.01 | 0.935 | 0.198 | 2.34E-06 | 4.99E-05 | 22.29 |  |
| rs187013421 | 10757677 | G | A | 0.009 | 1.014 | 0.215 | 2.30E-06 | 4.99E-05 | 22.33 |  |

Abbreviation: SNP, single nucleotide polymorphism; EA, Effect allele; NEA, Non-effect allele; EAF, effect allele frequency; SE, standard error;

*^a^ R^2^* was calculated the following formula:(2×EAF×(1-EAF)×beta^2^)/[(2×EAF×(1-EAF)×beta^2^)+(2×EAF×(1-EAF)×N×SE^2^)],

where EAF is the effect allele frequency, beta is the estimated effect on urate. Ν is the sample size of the GWAS for the SNP-urate association and SE is the standard error of the estimated effect.

*^b^ F* statistic was calculated using the following formula: *R^2^*(N-2)/(1-*R^2^*), where *R^2^* is the proportion of variance in urate explained by each instrument and N is the sample size of the GWAS for the SNP-urate association.

^c^ SNPs associated with confounding factors were removed after searching LDlink.
