# Supplementary material for: Proton pump inhibitors use and the risk of osteoporosis and fractures: A two-sample Mendelian randomization study
Source: Medicine (Baltimore). 2026 Jul 24;105(30):e49964. doi: 10.1097/MD.0000000000049964 (PMC13406325; doi:10.1097/MD.0000000000049964)
Supplement: Supplementary file 3 [file medi-105-e49964-s003.docx]

Table S3 Characteristics of SNPs used as genetic instruments for rabeprazole

| SNP | Position | EA | NEA | EAF | SNP-Exposure association | | | R^2 a^ | F-statistic ^b^ | Confounders ^c^ |
| --- | --- | --- | --- | --- | --- | --- | --- | --- | --- | --- |
|  |  |  |  |  | Beta | SE | P value |  |  |  |
| rs234121 | 640924 | T | C | 0.176 | 0.305 | 0.066 | 3.83E-06 | 4.78E-05 | 21.35 |  |
| rs7560777 | 1444577 | A | T | 0.489 | 0.227 | 0.05 | 4.70E-06 | 4.61E-05 | 20.96 | Educational attainment |
| rs72950416 | 1587540 | A | G | 0.377 | 0.243 | 0.051 | 2.17E-06 | 4.97E-05 | 22.43 |  |
| rs4395245 | 1864641 | G | A | 0.383 | -0.249 | 0.051 | 1.15E-06 | 5.21E-05 | 23.66 | BMI ,Type 2 diabetes |
| rs145923107 | 1866901 | A | G | 0.044 | 0.614 | 0.128 | 1.62E-06 | 5.09E-05 | 23 |  |
| rs397937 | 2003966 | C | A | 0.212 | 0.32 | 0.062 | 2.19E-07 | 6.00E-05 | 26.85 | Educational attainment |
| rs13098498 | 2748386 | T | C | 0.423 | 0.238 | 0.05 | 2.06E-06 | 4.94E-05 | 22.54 |  |
| rs187032336 | 3361447 | A | G | 0.01 | 1.33 | 0.284 | 2.86E-06 | 4.81E-05 | 21.91 |  |
| rs145956163 | 6219304 | A | G | 0.018 | 0.924 | 0.2 | 4.08E-06 | 4.68E-05 | 21.23 |  |
| rs10756389 | 6653412 | A | G | 0.203 | -0.294 | 0.064 | 3.69E-06 | 4.92E-05 | 21.42 |  |
| rs118182511 | 7844033 | C | G | 0.041 | 0.661 | 0.133 | 6.16E-07 | 5.50E-05 | 24.86 | BMI |
| rs142909537 | 10127375 | G | A | 0.007 | 1.553 | 0.336 | 3.80E-06 | 4.69E-05 | 21.36 |  |
| rs72810186 | 10330168 | G | T | 0.109 | 0.393 | 0.081 | 1.19E-06 | 5.21E-05 | 23.6 |  |

Abbreviation: SNP, single nucleotide polymorphism; EA, Effect allele; NEA, Non-effect allele; EAF, effect allele frequency; SE, standard error; BMI, body mass index.

*^a^ R^2^* was calculated the following formula:(2×EAF×(1-EAF)×beta^2^)/[(2×EAF×(1-EAF)×beta^2^)+(2×EAF×(1-EAF)×N×SE^2^)],

where EAF is the effect allele frequency, beta is the estimated effect on urate. Ν is the sample size of the GWAS for the SNP-urate association and SE is the standard error of the estimated effect.

*^b^ F* statistic was calculated using the following formula: *R^2^*(N-2)/(1-*R^2^*), where *R^2^* is the proportion of variance in urate explained by each instrument and N is the sample size of the GWAS for the SNP-urate association.

^c^ SNPs associated with confounding factors were removed after searching LDlink.
