# Supplementary material for: Proton pump inhibitors use and the risk of osteoporosis and fractures: A two-sample Mendelian randomization study
Source: Medicine (Baltimore). 2026 Jul 24;105(30):e49964. doi: 10.1097/MD.0000000000049964 (PMC13406325; doi:10.1097/MD.0000000000049964)
Supplement: Supplementary file 4 [file medi-105-e49964-s004.docx]

Table S4 Characteristics of SNPs used as genetic instruments for rabeprazole

| SNP | Position | EA | NEA | EAF | SNP-Exposure association | | | R^2 a^ | F-statistic ^b^ | Confounders ^c^ |
| --- | --- | --- | --- | --- | --- | --- | --- | --- | --- | --- |
|  |  |  |  |  | Beta | SE | P value |  |  |  |
| rs77410397 | 99879 | A | G | 0.155 | 0.075 | 0.016 | 1.94E-06 | 4.96E-05 | 22.65 |  |
| rs138655955 | 252887 | T | C | 0.036 | 0.164 | 0.031 | 1.28E-07 | 6.26E-05 | 27.89 |  |
| rs150445630 | 900535 | T | C | 0.005 | 0.418 | 0.087 | 1.47E-06 | 5.13E-05 | 23.19 |  |
| rs541096318 | 1330269 | C | A | 0.013 | 0.271 | 0.052 | 2.35E-07 | 6.10E-05 | 26.72 |  |
| rs11674978 | 1421461 | A | G | 0.419 | 0.053 | 0.012 | 4.57E-06 | 4.63E-05 | 21.01 |  |
| rs828618 | 2359642 | A | G | 0.291 | 0.058 | 0.013 | 3.86E-06 | 4.70E-05 | 21.33 |  |
| rs569327140 | 2834172 | C | T | 0.03 | -0.159 | 0.034 | 3.24E-06 | 4.98E-05 | 21.67 |  |
| rs72706312 | 3574074 | G | A | 0.09 | -0.094 | 0.02 | 4.32E-06 | 4.82E-05 | 21.12 |  |
| rs144574721 | 4359964 | G | A | 0.055 | -0.116 | 0.025 | 3.93E-06 | 4.68E-05 | 21.3 |  |
| rs2523589 | 4589769 | T | G | 0.495 | 0.059 | 0.011 | 2.35E-07 | 5.86E-05 | 26.72 | Type 2 diabetes |
| rs1555686 | 4617842 | G | A | 0.284 | -0.061 | 0.013 | 3.70E-06 | 5.16E-05 | 21.42 |  |
| rs79807832 | 4788429 | C | T | 0.027 | 0.17 | 0.035 | 1.71E-06 | 5.04E-05 | 22.9 | BMI |
| rs146833023 | 5812375 | T | C | 0.011 | 0.276 | 0.055 | 6.54E-07 | 5.48E-05 | 24.75 |  |
| rs7790767 | 5887559 | A | G | 0.008 | 0.307 | 0.066 | 3.55E-06 | 4.74E-05 | 21.49 |  |
| rs2386125 | 6466621 | C | T | 0.411 | 0.057 | 0.012 | 1.00E-06 | 5.25E-05 | 23.92 |  |
| rs10995788 | 7316496 | A | G | 0.228 | 0.066 | 0.014 | 1.30E-06 | 5.16E-05 | 23.42 |  |
| rs696178 | 7509085 | T | G | 0.214 | 0.066 | 0.014 | 2.22E-06 | 4.96E-05 | 22.39 | BMI |
| rs1890940 | 9475575 | C | T | 0.491 | -0.053 | 0.011 | 3.81E-06 | 4.68E-05 | 21.36 | BMI, Smoking initiation |
| rs62052427 | 10283371 | T | G | 0.278 | -0.059 | 0.013 | 4.06E-06 | 4.80E-05 | 21.24 |  |
| rs1842467 | 10925174 | T | C | 0.359 | 0.056 | 0.012 | 3.71E-06 | 4.90E-05 | 21.41 |  |
| rs117570374 | 11081612 | T | C | 0.044 | 0.128 | 0.028 | 4.23E-06 | 4.66E-05 | 21.16 | Educational attainment |
| rs10415242 | 11194290 | A | G | 0.086 | 0.099 | 0.021 | 1.80E-06 | 5.25E-05 | 22.79 |  |
| rs117842899 | 11785210 | C | G | 0.035 | 0.148 | 0.031 | 2.43E-06 | 4.99E-05 | 22.22 | Educational attainment |

Abbreviation: SNP, single nucleotide polymorphism; EA, Effect allele; NEA, Non-effect allele; EAF, effect allele frequency; SE, standard error; BMI, body mass index.

*^a^ R^2^* was calculated the following formula:(2×EAF×(1-EAF)×beta^2^)/[(2×EAF×(1-EAF)×beta^2^)+(2×EAF×(1-EAF)×N×SE^2^)],

where EAF is the effect allele frequency, beta is the estimated effect on urate. Ν is the sample size of the GWAS for the SNP-urate association and SE is the standard error of the estimated effect.

*^b^ F* statistic was calculated using the following formula: *R^2^*(N-2)/(1-*R^2^*), where *R^2^* is the proportion of variance in urate explained by each instrument and N is the sample size of the GWAS for the SNP-urate association.

^c^ SNPs associated with confounding factors were removed after searching LDlink.
