# Supplementary figures and images for: Proton pump inhibitors use and the risk of osteoporosis and fractures: A two-sample Mendelian randomization study
Source: Medicine (Baltimore). 2026 Jul 24;105(30):e49964. doi: 10.1097/MD.0000000000049964 (PMC13406325; doi:10.1097/MD.0000000000049964)

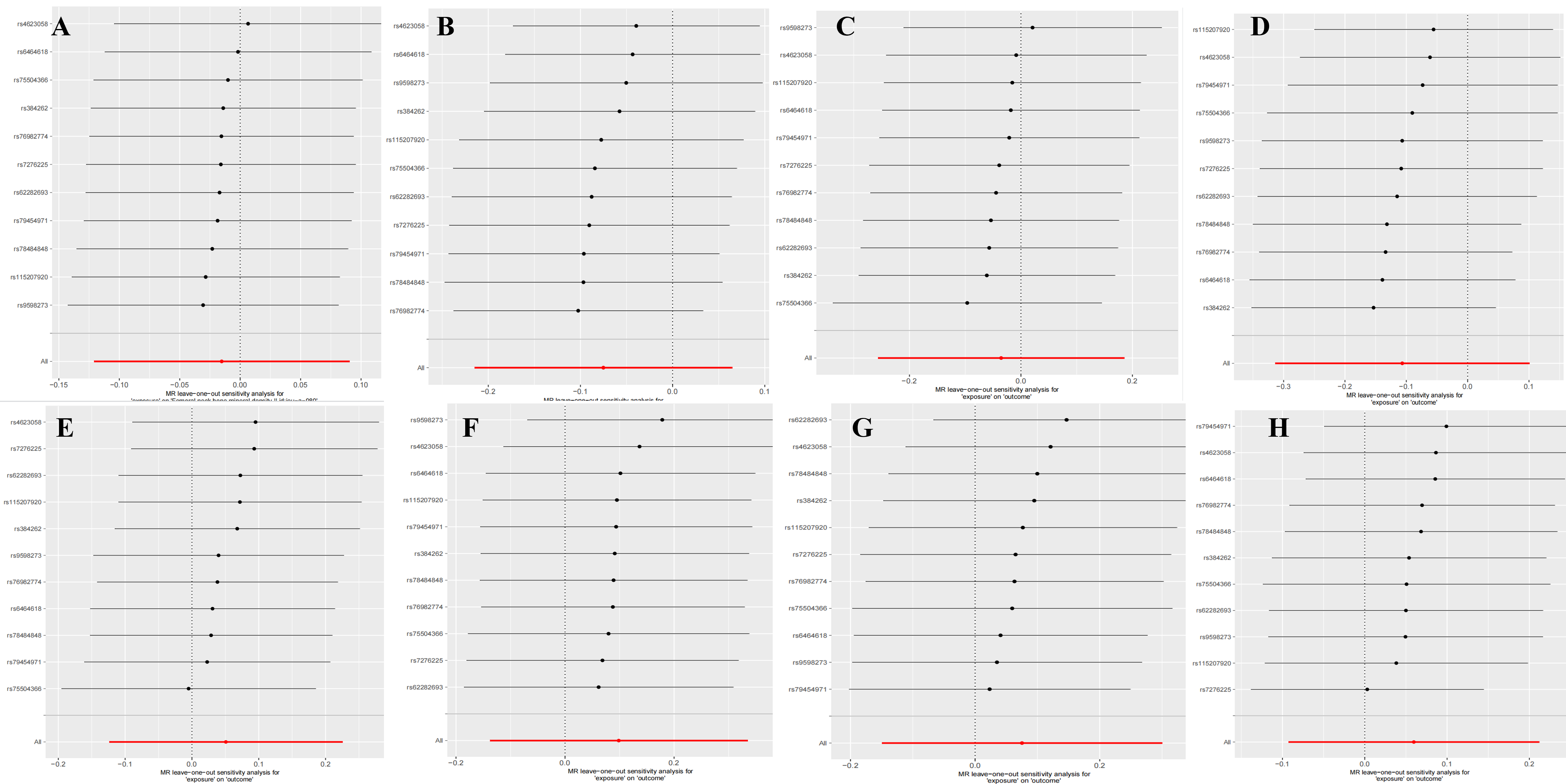

Supplement: Supplementary file 5 [file medi-105-e49964-s005.tiff]

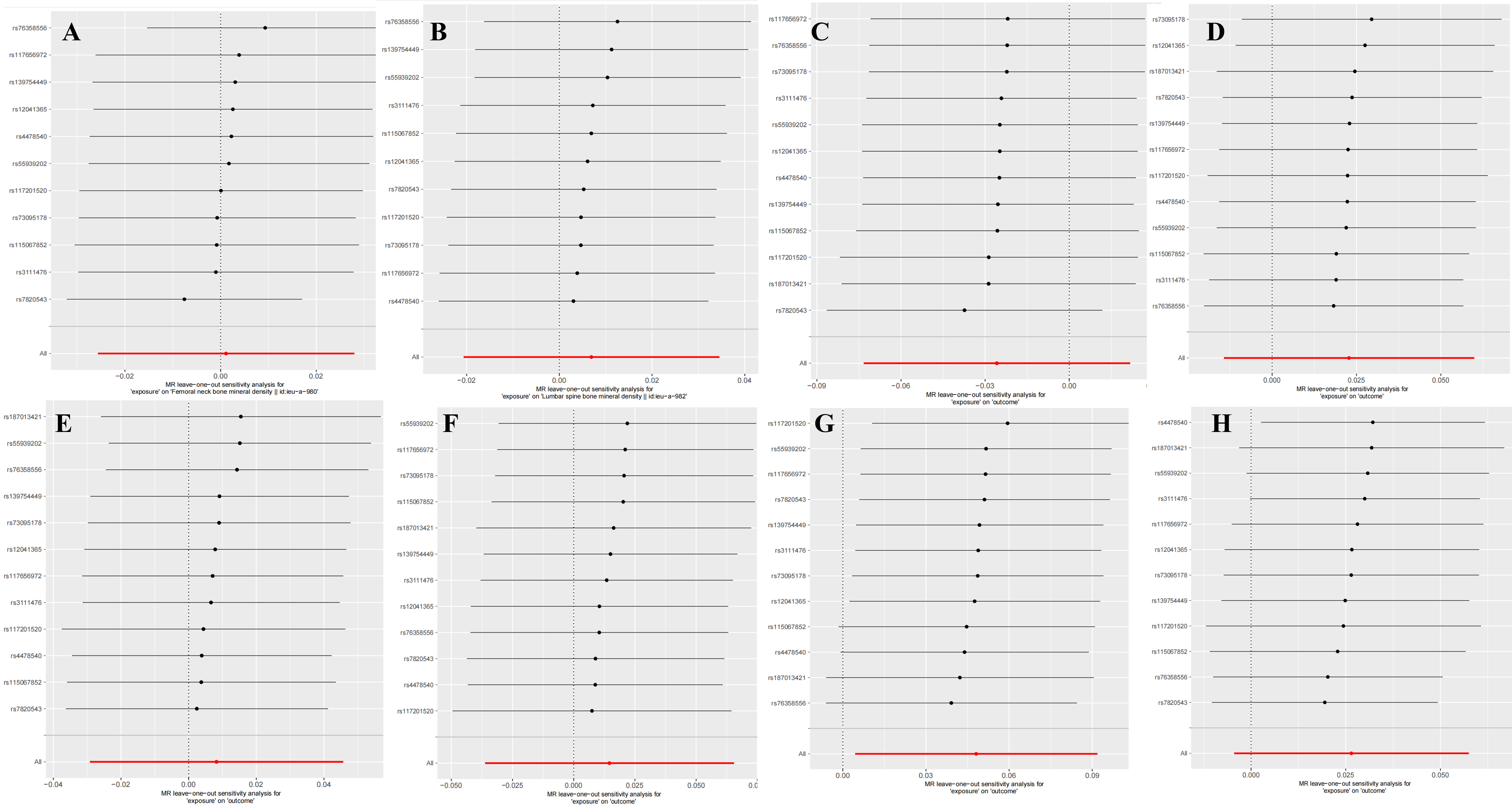

Supplement: Supplementary file 6 [file medi-105-e49964-s006.tiff]

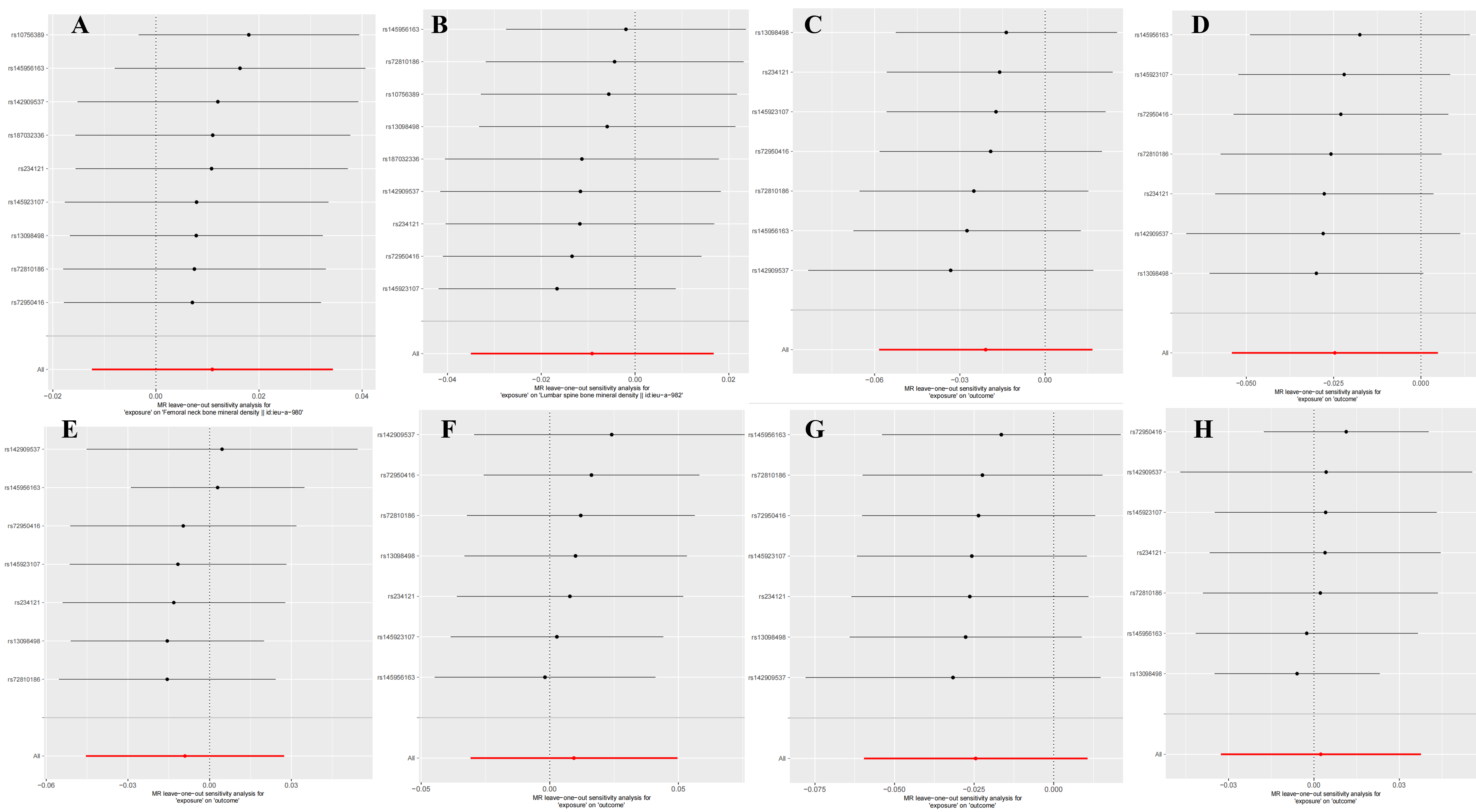

Supplement: Supplementary file 7 [file medi-105-e49964-s007.tiff]

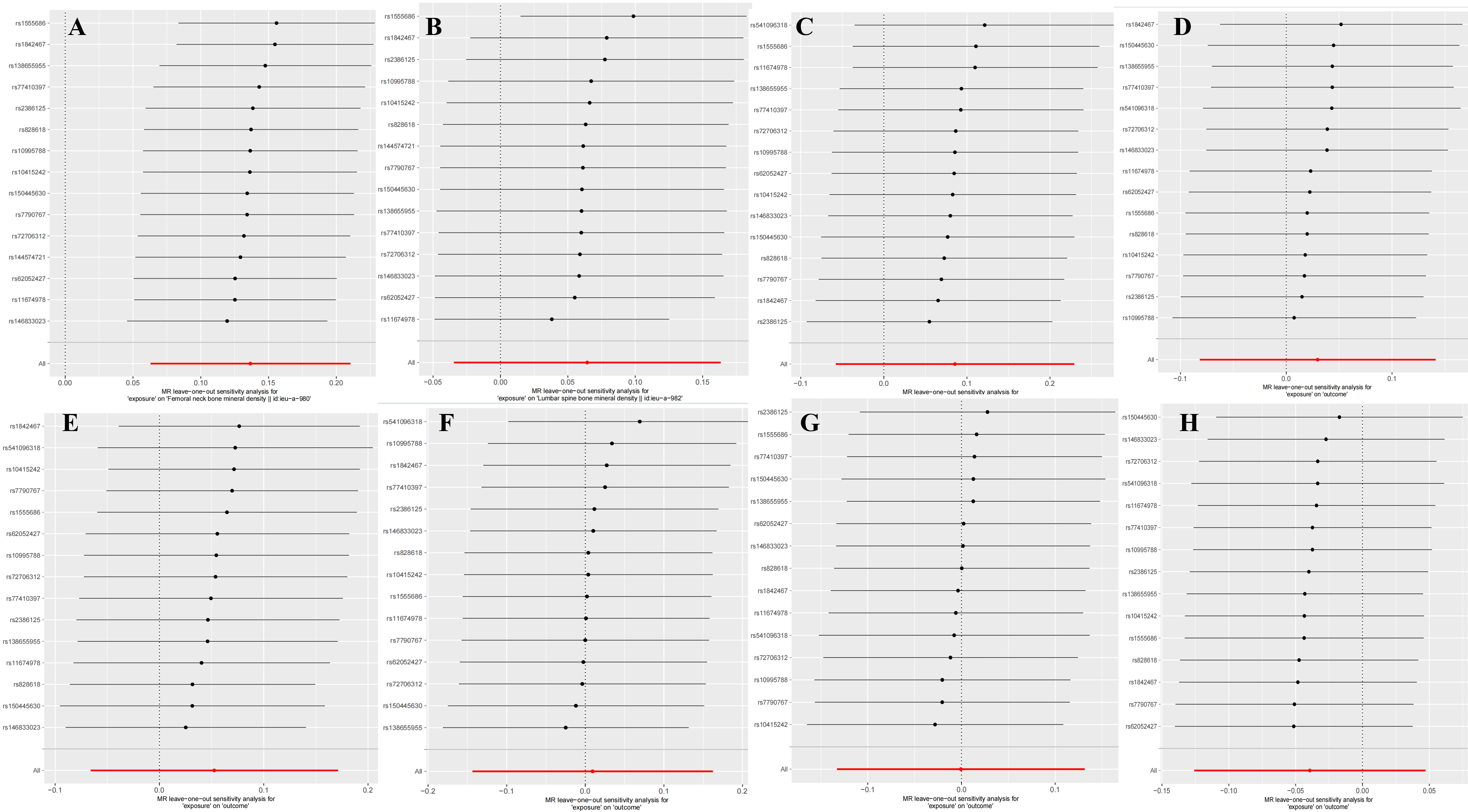

Supplement: Supplementary file 8 [file medi-105-e49964-s008.tiff]
